# Neuronal ceroid lipofuscinosis in the South American-Caribbean region: An epidemiological overview

**DOI:** 10.3389/fneur.2022.920421

**Published:** 2022-08-12

**Authors:** Guillermo Guelbert, Ana Clara Venier, Ines Adriana Cismondi, Adriana Becerra, Juan Carlos Vazquez, Elmer Andrés Fernández, Ana Lucía De Paul, Norberto Guelbert, Ines Noher, Favio Pesaola

**Affiliations:** ^1^Programa de Investigación Translacional de Lipofuscinosis Ceroidea Neuronal (NCL Program), Hospital de Niños de la Santísima Trinidad, Córdoba, Argentina; ^2^Servicio de Enfermedades Metabólicas Hereditarias, Hospital de Niños de la Santísima Trinidad, Córdoba, Argentina; ^3^Centro de Microscopía Electrónica, Facultad de Ciencias Médicas, Universidad Nacional de Córdoba, Córdoba, Argentina; ^4^Instituto de Investigación en Ciencias de la Salud, Consejo Nacional de Investigaciones Científicas y Técnicas, Córdoba, Argentina; ^5^Facultad de Odontología, Universidad Nacional de Córdoba, Córdoba, Argentina; ^6^Centro de Investigación y Desarrollo en Inmunología y Enfermedades Infecciosas, Universidad Católica de Córdoba, Consejo Nacional de Investigaciones Científicas y Técnicas, Córdoba, Argentina; ^7^Servicio de Enfermedades Metabólicas Hereditarias, Clínica Universitaria “Reina Fabiola”, Córdoba, Argentina; ^8^Universidad Nacional de Córdoba, Córdoba, Argentina; ^9^Department of Pediatrics, Washington University in Saint Louis School of Medicine, St. Louis, MO, United States

**Keywords:** neuronal ceroid lipofuscinoses (NCL), South America-Caribbean, epidemiology, genotype, phenotype

## Abstract

Neuronal ceroid lipofuscinoses (NCLs) comprise 13 hereditary neurodegenerative pathologies of very low frequency that affect individuals of all ages around the world. All NCLs share a set of symptoms that are similar to other diseases. The exhaustive collection of data from diverse sources (clinical, genetic, neurology, ophthalmology, etc.) would allow being able in the future to define this group with greater precision for a more efficient diagnostic and therapeutic approach. Despite the large amount of information worldwide, a detailed study of the characteristics of the NCLs in South America and the Caribbean region (SA&C) has not yet been done. Here, we aim to present and analyse the multidisciplinary evidence from all the SA&C with qualitative weighting and biostatistical evaluation of the casuistry. Seventy-one publications from seven countries were reviewed, and data from 261 individuals (including 44 individuals from the Cordoba cohort) were collected. Each NCL disease, as well as phenotypical and genetic data were described and discussed in the whole group. The CLN2, CLN6, and CLN3 disorders are the most frequent in the region. Eighty-seven percent of the individuals were 10 years old or less at the onset of symptoms. Seizures were the most common symptom, both at onset (51%) and throughout the disease course, followed by language (16%), motor (15%), and visual impairments (11%). Although symptoms were similar in all NCLs, some chronological differences could be observed. Sixty DNA variants were described, ranging from single nucleotide variants to large chromosomal deletions. The diagnostic odyssey was probably substantially decreased after medical education activities promoted by the pharmaceutical industry and parent organizations in some SA&C countries. There is a statistical deviation in the data probably due to the approval of the enzyme replacement therapy for CLN2 disease, which has led to a greater interest among the medical community for the early description of this pathology. As a general conclusion, it became clear in this work that the combined bibliographical/retrospective evaluation approach allowed a general overview of the multidisciplinary components and the epidemiological tendencies of NCLs in the SA&C region.

## Introduction

Neuronal ceroid lipofuscinoses (NCLs) are rare inherited neurodegenerative disorders of all ages, clinically characterized by progressive loss of speech, vision, cognitive and motor skills, with refractory seizures and early death. Taken together, they are the most common cause of neurodegeneration in childhood (1), although adulthood phenotype has also been described (2). Morphologically, NCLs are characterized by the accumulation of undegraded lipoprotein lipofuscin-like material within lysosomes (1), which includes them in the group of lysosomal storage disorders. To date, thirteen NCL diseases have been described, named according to the affected gene (CLN1-CLN14 diseases, CLN9 disease was suggested and later removed) (3). All the proteins encoded by these genes were defined; however, the specific role of some of them, and how they lead to the lysosomal pathology, remain to be fully elucidated.

Individuals affected by an NCL have been described and studied throughout the world, especially in the Northern Hemisphere where most of the “classical” (CLN1, CLN2, CLN3, CLN4, and CLN10 diseases), as well as the “variant” NCLs (CLN5, CLN6, CLN7, CLN8, CLN11, CLN12, CLN13, and CLN14 diseases), were identified for the first time. For example, CLN5 and CLN8 were first described in Finland (4, 5), CLN6 among the Romany population, and in a big Costa Rican family (6–9), and CLN7 was found in a cohort of Turkish children (10). The information of these individuals was subsequently collected in massive repositories to support the growing number of cases, either specific for NCLs (such as the NCL Resource https://www.ucl.ac.uk/ncl-disease/, supported by the University College London and curated by Dr Sara Mole; and the DEM-CHILD registry, led by Dr Angela Schulz), or for diverse disorders (such as Orphanet https://www.orpha.net/consor/cgi-bin/index.php, and LOVD https://www.lovd.nl). This information is then used, for example, for delineating the natural history of a particular NCL disease, the mutation spectrum, the population level, etc. Later, these results can finally be used as controls or reference points to compare future cases (11–13), and for epidemiological purposes (12).

There is a relative imbalance between the Northern and Southern Hemisphere countries of NCL cases registered in public databases. This might be due to differences in the number of referral centers and research facilities, the development of a robust and efficient national health system, the possibility and time to get a precise diagnosis (“diagnostic odyssey”), the knowledge of the diseases and the registries by the treating physicians, among others. The knowledge of a disease can in turn be increased by some other factors, such as available therapies, the degree of medical education, advocacy activities of family organizations, and other socio-economic factors. Thus, the South American and the Caribbean (hereinafter, SA&C) populations, for example, appear underrepresented in the databases. In the present review, we seek to carry out a multidisciplinary and epidemiological update of the NCL information in SA&C, collecting published information from different medical specialities (neurology, pediatrics, radiology, medical genetics, morphology, enzymology, electrophysiology, etc.) to build a baseline for regional medical use, avoid the registration of repeated cases and find out the regional specificities. Ultimately, we expect to feed regional and international databases and overcome the diagnostic odyssey, misdiagnosis, and underdiagnosis of various countries in this ethnically heterogeneous region (14).

## Analysis methodology

A comprehensive bibliographic search was made in the four principal databases of scientific articles (PubMed https://pubmed.ncbi.nlm.nih.gov/, ScienceDirect https://www.sciencedirect.com/, Google Scholar https://scholar.google.com/, and SciELO https://www.scielo.org/), using as keywords “Neuronal ceroid lipofuscinosis,” the HGNC approved symbol for each NCL gene (e.g., *PPT1*), its most common alias (e.g., CLN1, INCL), and the name of each SA&C country. All articles matching the keywords and including at least one author with a SA&C affiliation were collected. From the first compilation, only those articles with references to affected individuals (such as clinical, biochemical, genetic, morphological studies, etc.) were retrieved and subsequently analyzed. The individuals corresponding to the Cordoba cohort (15), whether or not published, who have a precise diagnosis and complete clinical data, were also included. In these cases, each individual or caregiver signed an informed consent approved by the local ethical board [Inter-institutional Committee of Ethics in Health Research (CIEIS—*Polo Hospitalario*)] authorizing not only the extraction, manipulation, and analysis of their samples for diagnostic purposes but also the dissemination of clinical data ensuring the total anonymity of the individuals. Those subjects with precise references to previous reports were tracked to avoid or reduce the number of duplications. All the information available (clinical, biochemical, genetic, morphological) was collected and analyzed using an Excel datasheet.

## Bibliographical evidence

**Figure 1:**
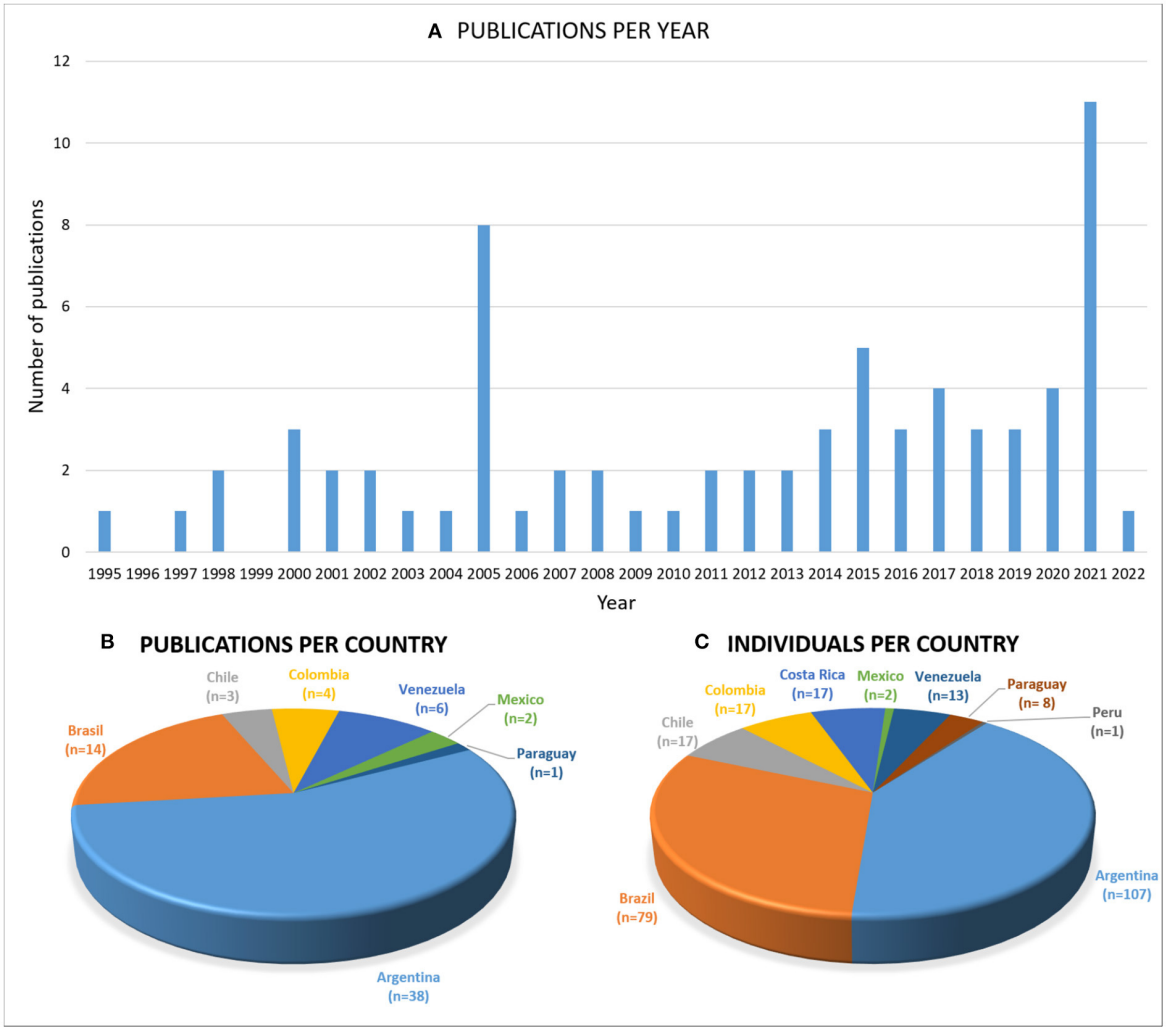
The SA&C publications. Graphs showing **(A)** the publications per year and **(B)** per country, and **(C)** the number of individuals described per SA&C country. All types of publications in public databases were collected. The country mentioned in affiliations was taken, even if the author from SA&C was not leading the article. An increasing number of publications (mainly on CLN2) was observed in the last few years. Argentina and Brazil are the most represented regarding the number of publications and individuals described, probably due to the increasing number of professionals and centers specialized in these pathologies.

Seventy-one articles published between 1995 and 2022 were collected from the four main literature databases (PubMed, Google Scholar, ScienceDirect, and SciELO), including original articles, reviews, case reports, short communications, and published conference abstracts (a complete list of this bibliography can be found in Supplementary Table 1). It should be noted that an attempt has been made to collect all the information published in public and massive bibliographic portals. Articles published in regional or local journals, conference presentations, or other non-print materials may have been omitted. It is important to highlight the importance of freely sharing clinical cases of rare diseases in public databases for a better understanding of these diseases. The number of publications has had “ups and downs” throughout the period considered with a significant increase in the last decade (Figure 1A). The first publication was by Taratuto et al. (16), in which they presented a group of late infantile NCL (LINCL) and juvenile NCL (JNCL) cases in Argentina. In 2005, a symposium book published by the National University Cordoba (Cordoba, Argentina), and edited by members of the recently formed NCL Program (established at the Children's Hospital of the Province of Cordoba in 2003), conducted an update of the NCL information derived mainly from research groups in SA&C. Individuals from different SA&C countries coursing with any NCL disease were described there, causing the publications peak observed in Figure 1A.

Authors from seven countries (Argentina, Brazil, Chile, Colombia, Mexico, Paraguay, and Venezuela) have collaborated in the publications reviewed (Figure 1B). Moreover, 77% of the articles were led by authors from SA&C. Individuals affected by an NCL were described in Argentina, Brazil, Chile, Colombia, Costa Rica, Mexico, Paraguay, Peru, and Venezuela (Figure 1C). Argentina (*n* = 38 articles) and Brazil (*n* = 17 articles) were the most represented countries, considering both the publications and the individuals described, reflecting the awareness of rare disease studies in the reference centers of these countries in the region.

## The SA&C cohort

### Phenotypic, genetic, and demographic distribution of cases

Two hundred sixty-one subjects affected by CLN1, CLN2, CLN3, CLN5, CLN6, CLN7, CLN8, CLN11, or CLN12 diseases were described in the literature and incorporated in this review, including some non-published cases of the Cordoba cohort (a complete list of these individuals and their associated medical information is presented in Supplementary Tables 2, 3). In older publications (16–23) the individuals were recorded according to clinical and/or morphological data [presence of intracellular bodies observed by transmission electron microscopy (TEM)], resulting in clinical definitions (e.g., Santavuori-Haltia disease or Jansky-Bielschowsky disease, later redefined as CLN1 and CLN2 diseases, respectively; infantile NCL [INCL], LINCL, JNCL). As genetic testing increased, these clinical definitions were correlated with genetic variants. Thus, individuals with an INCL phenotype were mostly diagnosed with CLN1, LINCL cases mostly with CLN2, and JNCL cases mostly with CLN3 disease. However, the genotype/phenotype correlation is not bidirectional (3). The current nomenclature and classification of NCLs was agreed by an international panel of experts in the clinical, genetic, biological, and morphological fields, and formally established in 2012 (24). It establishes the diagnostic definition of a case taking into account 7 axes (gene, genetic variant, biochemical phenotype, clinical phenotype, ultrastructural phenotype, functionality and other characteristics), which allows a precise definition in light of the heterogeneity of this group of pathologies. Because molecular confirmation was not performed in these old cases, the precise genetic variants affecting these children were not defined, so they were classified in this review as separated entities (NCL, INCL, LINCL, and JNCL in Supplementary Tables 2 , 3).

**Figure 2:**
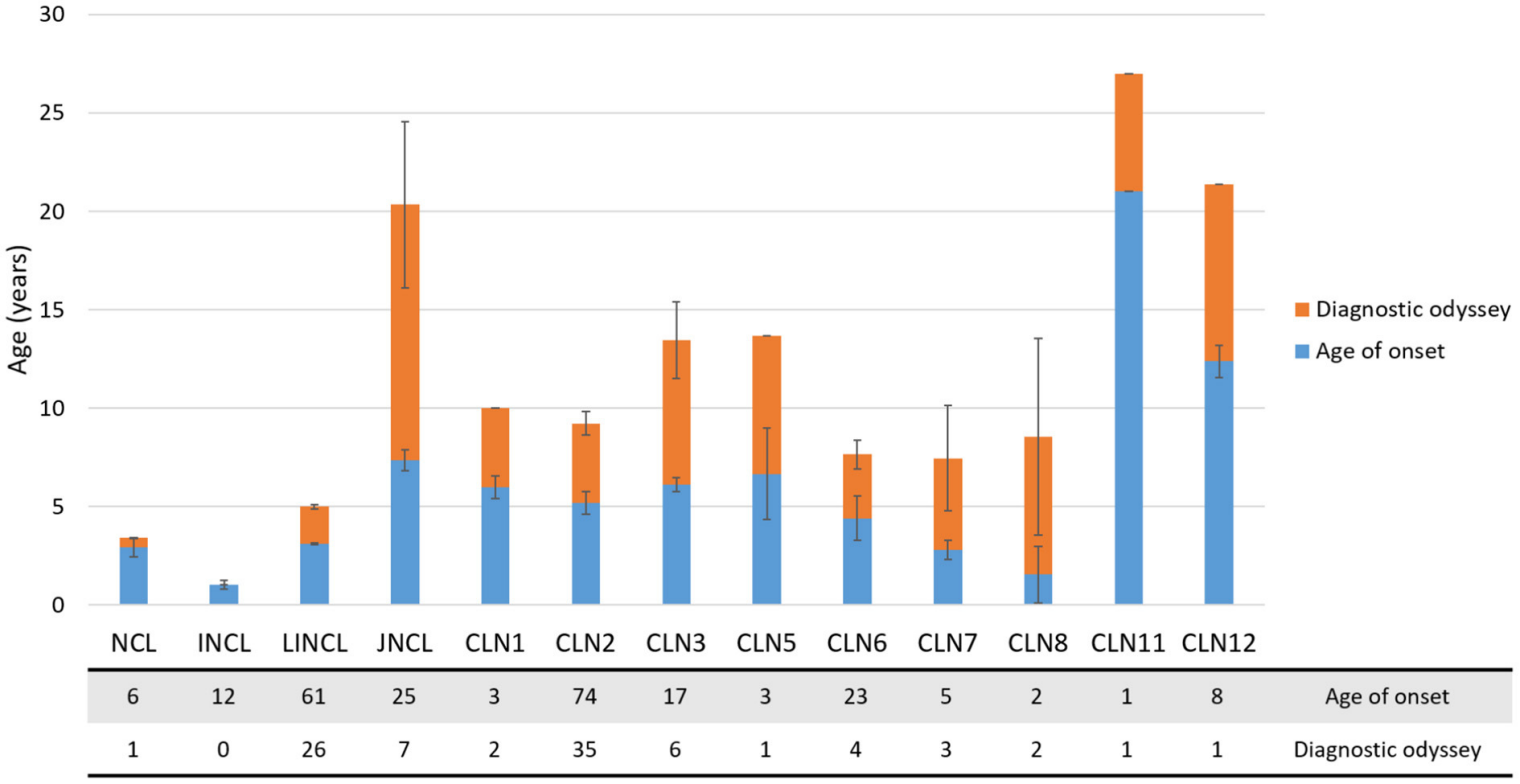
Diagnostic odyssey in the SA&C cohort. Bar graph showing the age of onset (mean ± SEM, blue bars) and time to diagnosis (mean ± SEM, orange bars) in the SA&C population analyzed. The age of onset could be recorded for 240 individuals, while the age at diagnosis could only be defined for 89 individuals. The table below the graph indicates de number of individuals included in each analyzed group.

Ninety-one individuals (35%) of the SA&C cohort are female, 77 are male (29%), and 93 (36%) were not identified. As expected, these values do not differ substantially from the estimated percentages of both genders in SA&C (males: 49.4%, females: 50.6%: Countrymeters https://countrymeters.info/es/South_America,revisedJune26,2022 ; Supplementary Table 3). In addition, 87% of the affected individuals were 10 years old or less at the onset of symptoms (Figure 2). Only those individuals affected by CLN11 or CLN12 disorders were older than 10 years old at the onset of symptoms. However, one individual affected by CLN12 disease was reported as having developmental delay since birth, although associated with perinatal hypoxia (25).

CLN2 disease is the most represented NCL in the region (*n* = 91), followed by CLN6 (*n* = 25) and CLN3 (*n* = 17). Likewise, LINCL (*n* = 77) and JNCL (*n* = 25) were the most abundant phenotypes. Unlike CLN2 cases, which were described along the subcontinent, other variants were observed as local concentrations. For example, an important cluster of CLN6 disease was described in Costa Rica (8, 26), two CLN8 cases in Argentina (27, 28), one CLN11 case in Brazil (29), and one and seven CLN12 cases in Brazil and Chile, respectively (Supplementary Table 2) (25, 30, 31). The CLN11 and CLN12 cases were not directly linked to an NCL. Instead, they were mostly referred to as frontotemporal lobar degeneration (caused by variants in the *GRN/CLN11* gene) or a Parkinson-like neurodegenerative syndrome caused by variants in the *ATP13A2/CLN12* gene. These genetic forms (as well as CLN13 and CLN14) were officially included in the NCL group in 2012, after confirming the presence of lipofuscin-like bodies in the cells of affected individuals (24). However, it is still very common to find them in the literature with the names of their clinical forms, referring to their association with NCLs as an annexe.

### The diagnostic odyssey

The time from the onset of the symptoms to the precise diagnosis did not vary significantly among the confirmed disorders (CLN1-CLN12; Figure 2). The average time to a precise diagnosis was 5.2 ± 1.0 years (mean ± SEM, ranging from 0 to 13 years). Some individuals were diagnosed in a short time because of either a genetic examination or having affected siblings. On the other hand, a prolonged time to diagnosis could be attributed to poor knowledge of these kinds of disorders, particularly before the availability of reliable genetic tests. In some cases (such as in CLN12), the diagnosis was made *post-mortem* (25).

### Clinical features

**Figure 3:**
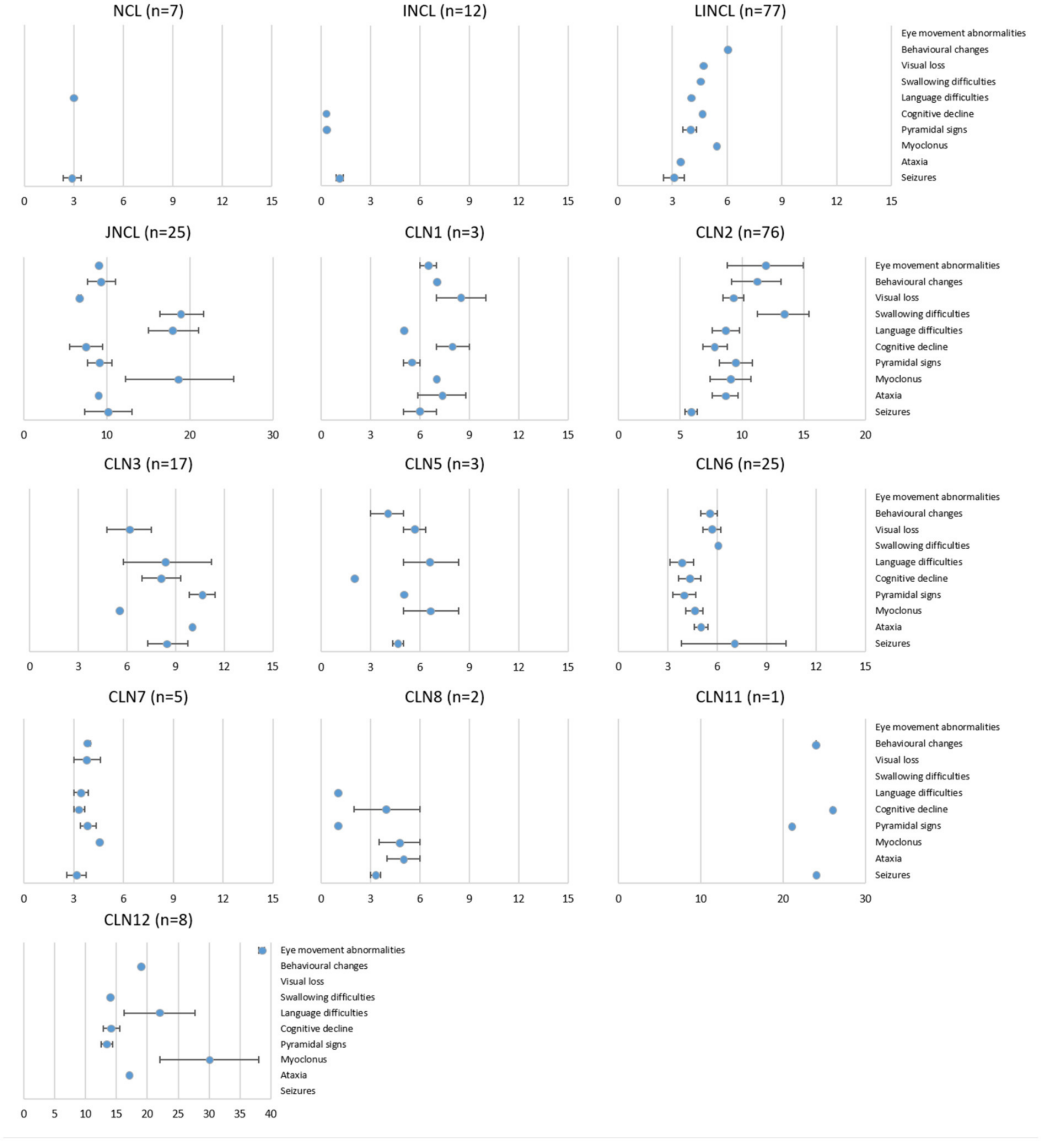
Disease evolution of all NCL variants in the SA&C cohort. Graphs showing the age of onset (mean ± SEM) of some symptoms for each NCL genotype and phenotype in the SA&C cohort. Values were obtained directly from clinical records or publications or estimated from the information available in the bibliography. The set of symptoms collected was adapted from Lourenço et al. (11). The number of individuals analyzed for each NCL variant is shown next to the graph titles. Values of the X-axis are expressed in years.

It is widely known that all NCLs share several clinical symptoms, such as seizures, psychomotor decline, and visual failure, which are also present in many other disorders (32). However, the chronological order in which they appear is usually considered at the time of differential diagnosis. The age of onset of different symptoms (seizures, ataxia, motor and cognitive deterioration, behavioral changes, and language and visual failure) was collected and analyzed for the entire SA&C cohort (Figure 3). The most common symptom at onset were seizures (51% of individuals) followed by language disorder (16%), motor impairment (15%), and visual failure (11%; Supplementary Figure 1). It should be noted that many times seizures are the symptom that leads to the first medical consultation, but not the first of the disorder. For example, some cognitive decline could be evident from an early age but attributed to other factors and dismissed. Seizures were also the most common symptom among all NCLs (12/13 groups), followed by pyramidal signs (11/13), cognitive decline (11/13), and language difficulties (10/13). The CLN6 and CLN7 disorders show a very rapid progression of symptoms, with very little variability between cases. Something similar occurs in CLN2 disease, although a greater variability in the onset of swallowing difficulties, behavioral changes and ocular abnormalities has been observed. It should be noted that in this review no discrimination has been made between those known as “classical” and “atypical” phenotypes. Thus, it should be considered that among the CLN2 cases there are “protracted” forms, with a slower symptomatic progression. On the other hand, we have observed a greater chronological dispersion of the symptoms in CLN3 cases (as in JNCL, although we cannot guarantee that they are all from the same genotype). Myoclonus has been observed at later ages in the CLN5, CLN7, CLN8, and CLN12 diseases. Vision loss occurs earlier in the CLN3 and CLN7 disorders. Language difficulties were described as appearing earlier in the CLN2 and CLN6 diseases. Finally, considering the ages of onset of symptoms, the reported cases of CLN1 in the SA&C region could be attributed more to a “protracted” than to a “classic” infantile phenotype (Figure 3).

All NCLs are characterized by the presence of lipofuscin-like intralysosomal bodies in all cells. These accumulations are observed by TEM in the form of defined patterns: granular osmiophilic deposits (GROD), curvilinear (CB), fingerprint (FP), or rectilinear bodies (RB), and can occur either alone or mixed (1). For a long time, the examination of tissue biopsies by TEM served as the method for the diagnosis of NCLs, leaving the genetic study for the definition of the particular disease. With the advent of new generation sequencing technologies, the genetic study is usually suggested within the main tests for diagnosis, reserving the morphological study only for challenging cases. More than half of the individuals in the SA&C cohort were analyzed by TEM (*n* = 159, 59%). The CB pattern was the most abundant among the individuals (*n* = 92, 35%), occurring more frequently in the LINCL (*n* = 59) and CLN2 groups (*n* = 20). On the other hand, the RB pattern has not been observed in the analyzed population (Supplementary Figure 2).

### The genetic background of the SA&C cohort

Sixty DNA variants have been described in 119 individuals of the SA&C population (Table 1; Supplementary Figure 3). One individual was reported to have a heterozygous variant in the *CLN6* gene without specifying it (39), and another with a positive linkage analysis pointing to chromosome 15, suggesting a variant in the *CLN6* gene (7). Seventy-seven percent have been variations in the coding sequence of the protein involved, mostly causing missense changes (42%). The intronic changes found (*n* = 13) correspond to splicing regions, having been their pathogenicity confirmed for the most frequent variant in the *TPP1/CLN2* gene, I7 c.887-10A>G, p.Pro295_Gly296insGluAsnPro (40) and the variant I13 c.1306+5G>A, p.Gly398_Leu435del in the *ATP13A2/CLN12* gene (25). Five deletions have been described, spanning few nucleotides [e.g., E9 c.1107_1108delTG, p.Gly370Lysfs^*^32 in the *TPP1/CLN2* gene (Cordoba cohort)], large intragenic deletions (such as the most frequent variant of CLN3 disease, 1.02 kb deletion, c.462_677del) (36, 41), or large chromosomal deletions (such as the deletion in the 8p23 region including the *CLN8* gene) (28). The pathogenicity of the variants has only been defined in 37% of cases. It should be noted that the pathogenicity of the variants found in the patients described is frequently not defined, or it is only bioinformatically. Experimental validation should be particularly necessary for changes whose effect on the protein is less obvious, such as missense changes or deep intronic variants in splicing regions. Although, on the other hand, the American College of Medical Genetics (ACMG) has proposed guidelines for the interpretation of variants that are currently widely used (42).

**Table 1:** List of DNA variants described in the SA&C cohort.

**Gene**	**Variant**	**Total alleles**	**Allelic frequency**	**Publications**
CLN1	E5 c.451C>T, p.Arg151* (P)	1	0.0019	Cordoba cohort
	I3 c.363-3T>G, p.? (U)	1	0.0019	Cordoba cohort
CLN2	E11 c.1424C>T, p.Ser475Leu (PP)	4	0.0077	Cordoba cohort, (11)
	E4 c.311T>A, p.Leu104* (P)	3	0.0057	Cordoba cohort
	I2 c.89+5G>C, p.? (U)	2	0.0038	Cordoba cohort
	E8 c.1048C>T, p.Arg350Trp (PP)	8	0.0153	Cordoba cohort, (11)
	I7 c.887-10A>G, p.Pro295_Gly296insGluAsnPro (P)	20	0.0383	Cordoba cohort, (11, 33)
	E7 c.827A>T, p.Asp276Val (P)	21	0.0402	Cordoba cohort, (11)
	I1 c.17+3G>T, p.? (U)	1	0.0019	Cordoba cohort
	E6 c.622C>T, p.Arg208* (P)	11	0.0211	Cordoba cohort, (11, 33)
	E8 c.1016G>A, p.Arg339Gln (PP)	2	0.0038	Cordoba cohort
	E3 c.196C>T, p.Gln66* (P)	5	0.0096	Cordoba cohort, (11)
	E11 c.1340G>A, p.Arg447His (PP)	6	0.0115	Cordoba cohort, (11, 34)
	E7 c.225G>A, p.Gln75Gln (P)	1	0.0019	(35)
	E11 c.1266G>C, p.Gln422His (PP)	1	0.0019	(11)
	E12 c.1438G>A, p.Val480Met (U)	6	0.0115	(11, 33)
	I8 c.1076-2A>T, p.? (PP)	10	0.0192	(11, 33)
	E11 c.1358C>T, p.Ala453Val (PP)	1	0.0019	Cordoba cohort
	E11 c.1358C>A, p.Ala453Asp (PP)	2	0.0038	Cordoba cohort
	E13 c.1603G>C, p.Gly535Arg (PP)	4	0.0077	Cordoba cohort, (11)
	E9 c.1107_1108delTG, p.Gly370Lysfs*32 (PP)	1	0.0019	Cordoba cohort
	I5 c.509-1G>C, p.? (PP)	1	0.0019	Cordoba cohort
	I12 c.1552-1G>A, p.? (PP)	2	0.0038	(11, 34)
	E6 c.503_504insTGGA, p.Phe169Glyfs*20 (P)	1	0.0019	(35)
	E12 c.1439T>G, p.Val480Gly (PP)	1	0.0019	(11)
	E11 c.1343C>A, p.Ala448Asp (PP)	1	0.0019	(11)
	E6 c.616C>T, p.Arg206Cys (P)	2	0.0038	(33)
	E5 c.471C>A, p.Tyr157* (PP)	1	0.0019	(33)
CLN3	E14 c.1195G>T, p.Glu399* (PP)	1	0.0019	Cordoba cohort
	1.02 kb deletion (c.462_677del)	10	0.0192	Cordoba cohort, (36)
	E6 c.400T>C, p.Cys134Arg (PP)	1	0.0019	Cordoba cohort
	E13 c.1000C>T, p.Arg334Cys (PP)	1	0.0019	Cordoba cohort
CLN5	E1 c.291_292insC, p.Ser98Leufs*13 (PP)	2	0.0038	Cordoba cohort
	E2 c.335G>A, p.Arg112His (P)	4	0.0077	(37)
CLN6	I4 c.486+8C>T, p.? (U)	1	0.0019	Cordoba cohort
	E4 c.307C>T, p.Arg103Trp (PP)	1	0.0019	Cordoba cohort
	E4 c.461_463delTCA, p.Ile153del (P)	1	0.0019	Cordoba cohort
	E6 c.662A>C, p.Tyr221Ser (PP)	2	0.0038	(8)
	E3 c.214G>T, p.Glu72* (P)	23	0.0441	(8, 26)
	E6 c.552_552delC, p.Phe185Serfs*21 (PP)	2	0.0038	(38)
	E5 c.510_512delCTA, p.Tyr171del (PP)	2	0.0038	(8)
	E7 c.755G>A, p.Arg252His (PP)	1	0.0019	Cordoba cohort
	E6 c.555_556insC, p.Phe186Leufs*16 (PP)	1	0.0019	Cordoba cohort
	E3 c.250T>A, p.Tyr84Asn (PP)	1	0.0019	Cordoba cohort
	E4 c.368G>A, p.Gly123Asp (P)	7	0.0134	(8)
	E7 c.722T>C, p.Met241Thr (P)	7	0.0134	(8)
	E3 c.244G>C, p.Gly82Arg (PP)	2	0.0038	(38)
	I2 c.198+104T>C (U)	16	0.0307	(26)
CLN7	I2 c.63-4delC, p.? (U)	2	0.0038	Cordoba cohort
	E3 c.103C>T, p.Arg35* (P)	5	0.0096	Cordoba cohort
	E12 c.1444C>T, p.Arg482* (P)	1	0.0019	Cordoba cohort
	I9 c.863+1G>A, p.? (PP)	1	0.0019	Cordoba cohort
CLN8	E2 c.1A>G, p.? (PP)	1	0.0019	Cordoba cohort
	Deletion of 378.6 kb in 8p23 region	2	0.0038	(28)
	E3 c.792C>G, p.Asn264Lys (P)	1	0.0019	Cordoba cohort
CLN11	E8 c.767_768insCC, p.Gln257Profs*27 (P)	2	0.0038	(29)
CLN12	E15 c.1510G>C, p.Gly504Arg (PP)	2	0.0038	(30)
	I22 c.2529+1G>A, p.? (P)	2	0.0038	(31)
	E26 c.3057_3057delC, p.Tyr1020Thrfs*3 (P)	7	0.0134	(25, 31)
	I13 c.1306+5G>A, p.Gly398_Leu435del (P)	5	0.0096	(25)

*The allelic frequency was calculated based on all the individuals in the SA&C cohort. P, pathogenic; PP, probably pathogenic; U, uncertain significance*.

When a rare genetic disorder with autosomal recessive inheritance is diagnosed, consanguinity between the parents is usually suspected. The consanguinity could only be confirmed in 10% (*n* = 25) of the population analyzed. However, it could not be defined in more than half of the individuals (57%), suggesting that a higher percentage might be observed (Supplementary Table 3).

Over time, the increase in the number of sequencing performed and their inclusion in the databases lead to an update of the consensus sequences. For example, the update of the human genome sequence toward version GRCh38.p14 (latest to date) was released on February 3, 2022. Although these updates do not usually cause major changes in the nomenclature of the variants described, it would be advisable to include in the publications the data of the version of the database used to identify and validate the variant (for example, the transcript identifier).

### Enzymatic tests in the SA&C region

Enzyme activity assays for PPT1 and TPP1 (also for CTSD, although its application is less common) are widely used as a rapid screening method for the CLN1 and CLN2 (and CLN10) diseases, respectively (43–45). However, other NCLs (such as CLN5, CLN6, CLN7, and CLN8) can also show reduced enzymatic values (and not only PPT1 and TPP1 but also all the lysosomal enzymes), thus providing data to guide the diagnosis (27, 46–49). For example, the proteins CLN6 and CLN8, present in the membrane of the endoplasmic reticulum, make up the EGRESS complex responsible for transporting soluble lysosomal enzymes to the Golgi apparatus. It has been shown that the deficiency of any of these proteins causes a decrease in the amount of soluble enzymes that reach the lysosome, thus generating a generalized lysosomal deficiency (46, 49). In the SA&C cohort, only 20% of individuals had PPT1 or TPP1 enzyme activity studied in any of the tissues used [leukocyte pellet, dried blood spot (DBS), or saliva; Supplementary Figure 4]. The most common assay has been the measurement of TPP1 activity in leukocyte pellets (20%) followed by the TPP1 activity assay in DBS (19%). There is a bias in these values toward the analysis of CLN2 disease in these samples. First, enzymatic analysis in leukocyte pellet is considered the “gold standard” for the diagnosis of both CLN1 and CLN2 diseases; therefore, it tends to be more frequently reported in publications (if these tests are performed in several tissues). On the other hand, DBS analysis is the most widespread worldwide, due to the practicality of sending samples over long distances with a minimum of deterioration. PPT1 and TPP1 activity assays in saliva were first described by Kohan et al. in 2005 (43). Despite being minimally invasive and with quantitative robustness like that obtained with the leukocyte pellet (50), its use has not reached the extent of other samples. Lastly, enzyme assays are not always the first option for NCL screening. When performing a genetic test and observing DNA variants in genes other than *PPT1/CLN1* or *TPP1/CLN2* (or *CTSD/CLN10*), enzymatic assays are not performed, and quite sensibly. In addition, results “within the reference interval” (i.e., within the range of control values) may be usually disregarded for final publication.

### Neuroimaging and electrophysiology studies

Certain features common to all NCLs were noted in the results of neurophysiological and imaging studies. Magnetic resonance images and computed tomography studies showed, to a greater or lesser extent, some degree of cerebral and/or cerebellar atrophy, with signal hyperintensity in periventricular regions also being very common. Electroencephalography studies generally showed slowing of basal rhythms, with focal or generalized epileptic paroxysms, with generalized polyspike or spike-wave phenomena. Electroretinogram and visual evoked potentials studies were performed mostly in those cases showing some degree of visual loss, observing optic nerve atrophy, retinitis pigmentosa, pale pupils, and thinning of retinal capillaries, among others. Supplementary Table 3 shows detailed information on the results of the studies performed on all patients in the SA&C cohort.

## Concluding remarks

Neuronal ceroid lipofuscinoses are a heterogeneous group of rare disorders sharing a handful of symptoms that, in turn, are common in other neurodegenerative pathologies. However, particular symptoms, as well as the sequential combination of them, can be recognized in NCLs, helping in some way to guide the diagnosis. To improve this, it is important to collect and study the set and sequence of phenotypic features of each precisely diagnosed NCL through its manifestation in each individual. The study of the SA&C population of affected individuals is in this sense a “black pearl” to delineate the clinical assessment of new cases. Although many subjects have been reported as coursing a “classical” natural history, many others have broken the “classical” forms introducing “atypical” symptoms or disease evolution to the spectrum of NCL phenotypes. Such are the cases of CLN2 (the “atypical” or “protracted” variant described in the Cordoba cohort) (51), CLN6 (Costa Rica's variant) (8, 26, 52, 53), and CLN8 diseases (the congenital variant) (27). This heterogeneity may be due to the ethnic and genetic diversity imprinted on the SA&C population, as suggested by some authors (50).

This review brings with it a series of limitations: the literature included was only that available in public databases; many relevant clinical data have not been reported in the publications, either due to omission, ignorance or were simply out of the scope of the work; the criteria for defining a non-obvious symptom may vary between different clinicians, leading sometimes to a late description of its onset: despite our efforts, some individuals were likely counted more than once in our analysis due to inefficient identification in the literature; and on the other hand, those cases that have not been published have been left out of this work (except for the cases of our research center). However, the complexity and quantity of the information collected allow us to address some points: (1) the multidisciplinary approach allowed us to describe and compare the evolution of each NCL in the region and to recognize some of the peculiarities of each genotype. Despite the phenotypic similarities between NCLs with each other and with other pathologies, there are certain variations (mainly chronological) that may guide medical diagnosis. Similarly, a multidisciplinary study (clinical, genetic, enzymatic, radiological, ophthalmological, etc.) of each particular case is always necessary; (2) Certain NCLs are more studied worldwide than others, such as CLN2 and CLN3. In principle, this may be since they are the most abundant NCLs, and therefore, the most important for the prompt search for effective treatments. However, the commercial availability of enzyme replacement therapy for CLN2 in 2017 has aroused medical interest in the early diagnosis of this pathology, and scientific interest in studying the results of its application. This led to an increase in published articles on this pathology, both worldwide and in SA&C; (3) The less prevalent phenotypes may still be underdiagnosed in many countries. Medical and technological advances promote awareness of some diseases, as happened with therapy for CLN2. This can pose two future scenarios: that the search for a “better known” disease leads to the diagnosis of another “less known,” or that the “less known” are underdiagnosed. Despite this, since NCLs are still little known to many health professionals, underdiagnosis may be generalized for all of them; (4) Knowledge about these rare diseases was increased in countries such as Argentina, Brazil, and Chile as an indicator of the impact of genomic technology, new therapeutic interventions based on enzyme replacement technology and gene therapy, medical education, and family advocacy; (5) The diagnostic odyssey gradually decreased (mainly in the most advanced countries of the region), probably as the diseases became better known by the local medical community after the appearance of new therapeutic solutions on the immediate horizon, as well as the earlier implementation of specific (panels of genes) or generalized genetic studies (genomic or exomic studies); (6) Diagnosis through TEM has been gradually replaced by genetic studies. Currently, the wide availability of genetic tests, as well as the minimal intervention on the patient (blood sample vs. tissue biopsy) has promoted this transition. However, since the accumulation of intralysosomal compounds is the pathognomonic feature of NCLs, this practice is suggested in cases where the clinic and genetics do not allow arriving at the same diagnosis.

In summary, an exhaustive search of the public literature on NCLs by SA&C authors, as well as referring to affected individuals in the same region, has been performed for this review. In the same way, the clinical information of 44 individuals included in the Cordoba cohort since 2003 has been compiled. Altogether, 71 scientific articles and 261 individuals affected by any NCL have been analyzed, becoming the largest compilation to date of clinical and bibliographic information on NCLs for SA&C. This work aims to promote the creation and/or improvement of public databases for the region, strengthen the information network on NCLs, lay the foundations for rigorous criteria for clinical data collection and help diagnose these challenging pathologies.

## Author contributions

GG contributed to clinical assessment, data collection, writing, and review. ACV contributed to data collection and review. IAC contributed to the planning and revision of the work. AB and NG contributed to the clinical assessment. JCV and EAF contributed to bioinformatics and review. ADP contributed to the review. IN contributed to data collection, the planning, and revision of the work. FP contributed to planning, data collection and analysis, writing, graphical formatting, and review. All authors contributed to the article and approved the submitted version.

## Funding

The NCL Program has received funding from the Consejo Nacional de Investigaciones Cientificas y Tecnicas (CONICET), Fondo para la Investigacion Cientifica y Tecnologica (FONCyT), Universidad Nacional de Cordoba (grant number 33620180100993CB to EF), Universidad Católica de Córdoba (grant number 80020180100029CC to EF), Hospital de Ni1os de la Santisima Trinidad de Cordoba, and Batten Disease Support and Research Association (BDSRA) for its operation for so many years.

## Conflict of interest

The authors declare that the research was conducted in the absence of any commercial or financial relationships that could be construed as a potential conflict of interest.

## Publisher's note

All claims expressed in this article are solely those of the authors and do not necessarily represent those of their affiliated organizations, or those of the publisher, the editors and the reviewers. Any product that may be evaluated in this article, or claim that may be made by its manufacturer, is not guaranteed or endorsed by the publisher.

## Supplementary material

Supplementary Figure 1 Onset symptoms in the SA&C cohort. Graph showing the symptoms (or set of them) and percentage of total individuals that showed them at the onset of the disorder. In the cases of individuals that showed more than one symptom at onset, they were added to each group. Seizures are significantly the most common symptom at onset (possibly overestimated) followed by language disorders, motor impairment and visual failure. The NA group represents those individuals with data not available.

Supplementary Figure 2 Lipofuscin-like accumulation in the SA&C cohort of individuals. Bar graph showing the percentage of individuals affected by each NCL disorder that showed any kind of lipofuscin-like accumulation observed by TEM. The total number of individuals in each NCL disease is shown on the X-axis. Curvilinear bodies (CB) are significantly the most represented pattern observed in most of the NCLs, followed by granular osmiophilic deposits (GROD) and fingerprints (FP). Rectilinear bodies (RB) were not observed in any of the individuals analyzed. Those individuals that showed more than one pattern (mixed) were added to all the corresponding groups.

Supplementary Figure 3 Summary of the DNA variants information in the SA&C cohort. Graphs showing information about the **(A)** position, **(B)** protein effect and **(C)** predicted consequence of all DNA variants described in the SA&C cohort. In those cases where the pathogenicity of the DNA variant was not defined in the publication, it was predicted bioinformatically by using Mutation Taster (https://www.mutationtaster.org/).

Supplementary Figure 4 Enzymatic analyzes in SA&C. Bar graphs showing the number of individuals analyzed enzymatically for each type of sample and NCL disorder. TPP1 was significantly more analyzed than PPT1 in all samples and NCLs. In addition, leukocytes and dried blood spots (DBS) are significantly more used than saliva. In turn, it is observed that the largest number of tests were performed for individuals affected by CLN2 disease, as expected. Likely, there is a bias mainly toward CLN2 disease on the total number of tests performed, due to the lack of information on enzyme assays in other NCLs. N, the total number of individuals analyzed for each NCL disorder. If an individual was analyzed for more than one tissue and/or enzyme, it was added to all the corresponding groups.
