# Supplementary figures and images for: Neuronal ceroid lipofuscinosis in the South American-Caribbean region: An epidemiological overview
Source: Front Neurol. 2022 Aug 12;13:920421. doi: 10.3389/fneur.2022.920421 (PMC9412946; doi:10.3389/fneur.2022.920421)

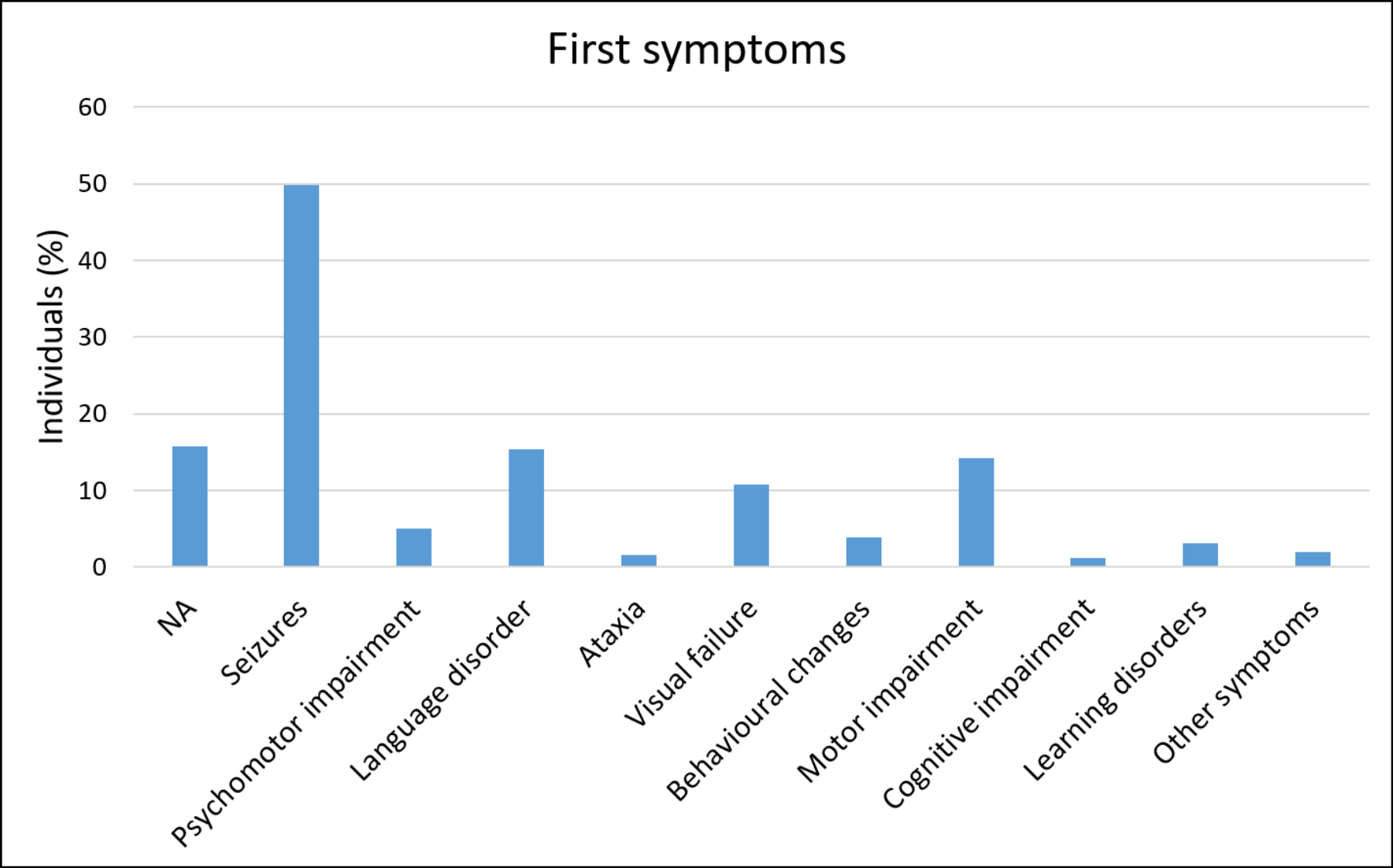

Supplement: Supplementary Figure 1 — Onset symptoms in the SA&C cohort. Graph showing the symptoms (or set of them) and percentage of total individuals that showed them at the onset of the disorder. In the cases of individuals that showed more than one symptom at onset, they were added to each group. Seizures are significantly the most common symptom at onset (possibly overestimated) followed by language disorders, motor impairment and visual failure. The NA group represents those individuals with data not available. [file Image_1.TIF]

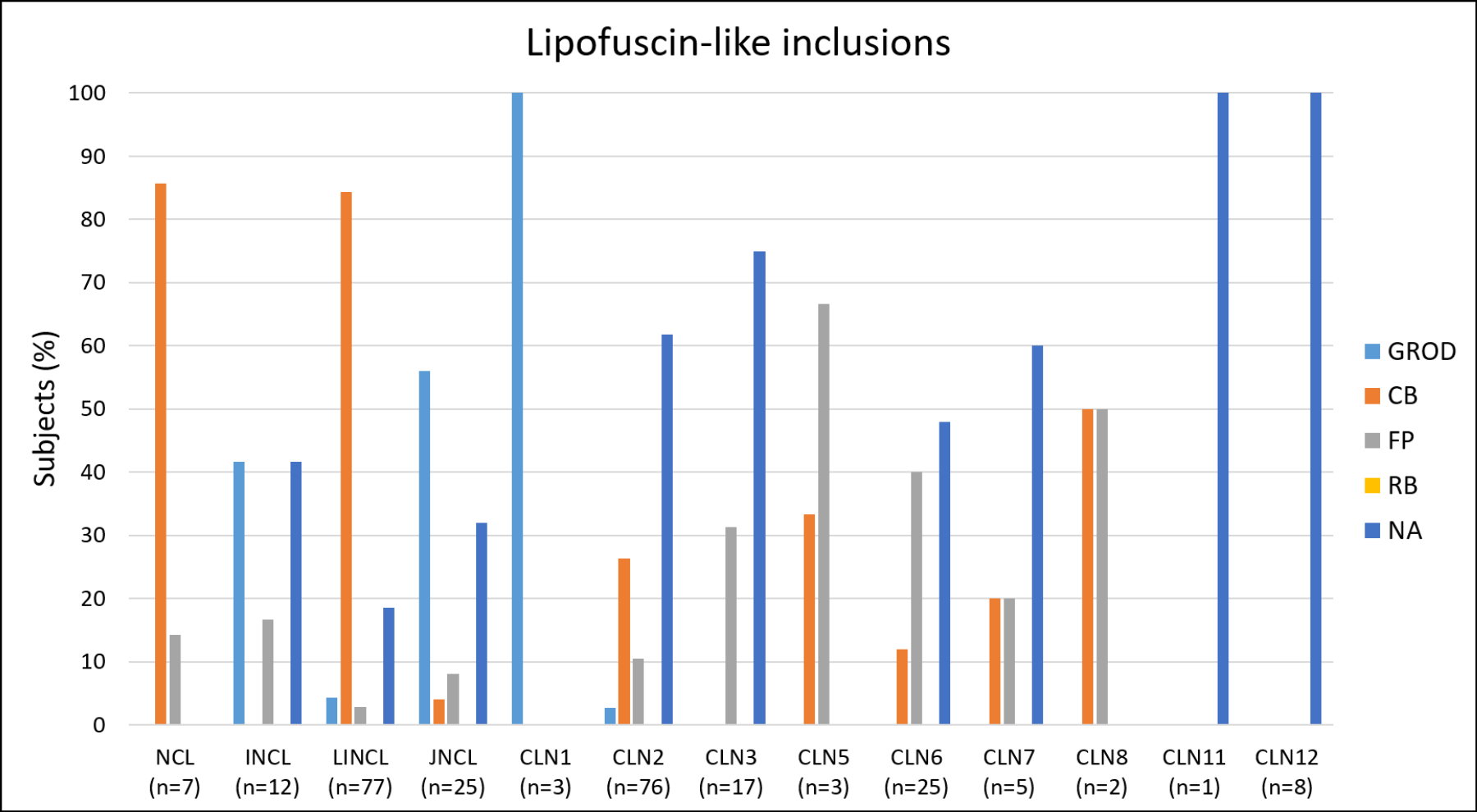

Supplement: Supplementary Figure 2 — Lipofuscin-like accumulation in the SA&C cohort of individuals. Bar graph showing the percentage of individuals affected by each NCL disorder that showed any kind of lipofuscin-like accumulation observed by TEM. The total number of individuals in each NCL disease is shown on the X-axis. Curvilinear bodies (CB) are significantly the most represented pattern observed in most of the NCLs, followed by granular osmiophilic deposits (GROD) and fingerprints (FP). Rectilinear bodies (RB) were not observed in any of the individuals analyzed. Those individuals that showed more than one pattern (mixed) were added to all the corresponding groups. [file Image_2.TIF]

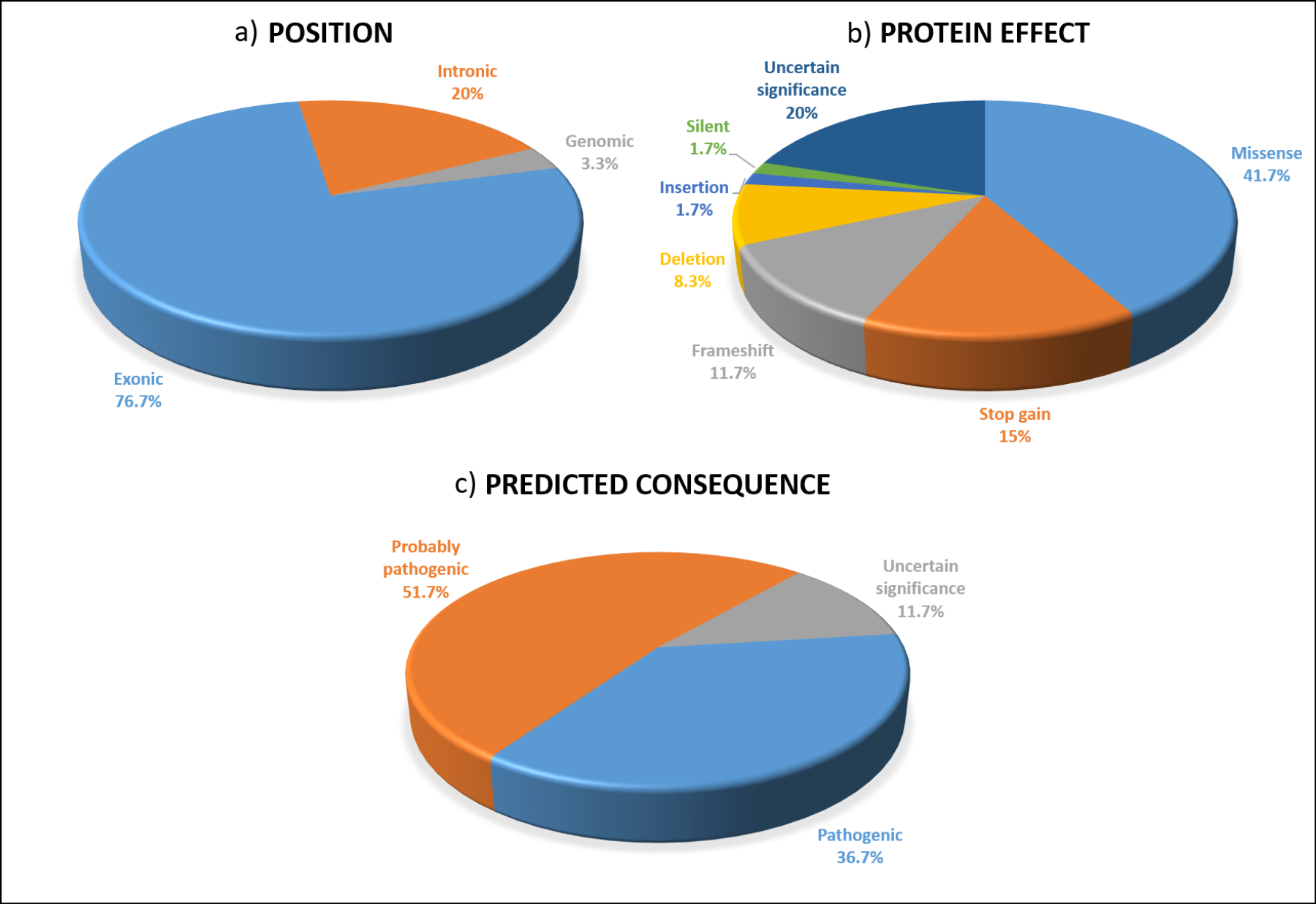

Supplement: Supplementary Figure 3 — Summary of the DNA variants information in the SA&C cohort. Graphs showing information about the (A) position, (B) protein effect and (C) predicted consequence of all DNA variants described in the SA&C cohort. In those cases where the pathogenicity of the DNA variant was not defined in the publication, it was predicted bioinformatically by using Mutation Taster (https://www.mutationtaster.org/). [file Image_3.TIF]

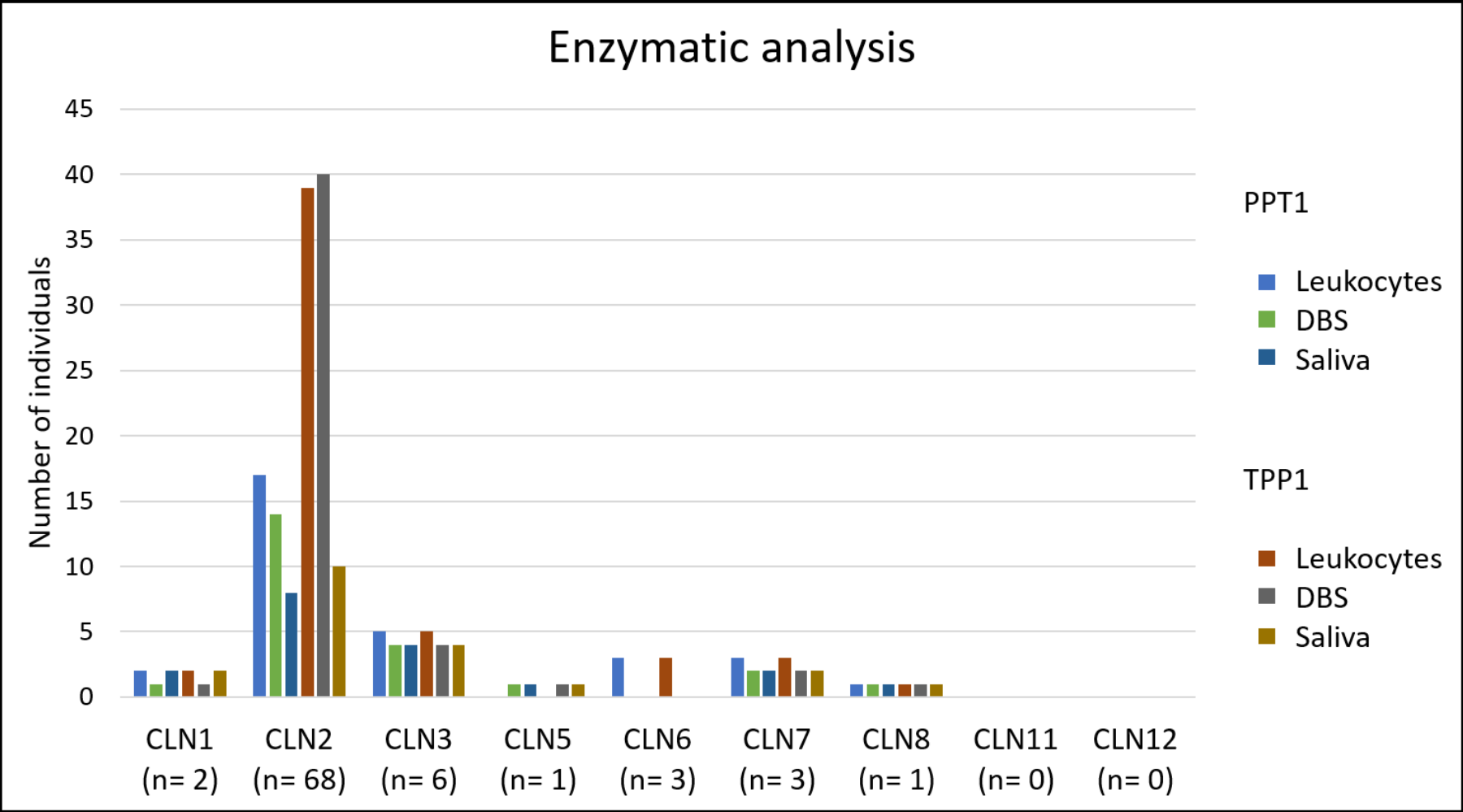

Supplement: Supplementary Figure 4 — Enzymatic analyzes in SA&C. Bar graphs showing the number of individuals analyzed enzymatically for each type of sample and NCL disorder. TPP1 was significantly more analyzed than PPT1 in all samples and NCLs. In addition, leukocytes and dried blood spots (DBS) are significantly more used than saliva. In turn, it is observed that the largest number of tests were performed for individuals affected by CLN2 disease, as expected. Likely, there is a bias mainly toward CLN2 disease on the total number of tests performed, due to the lack of information on enzyme assays in other NCLs. N, the total number of individuals analyzed for each NCL disorder. If an individual was analyzed for more than one tissue and/or enzyme, it was added to all the corresponding groups. [file Image_4.TIF]
