# Supplementary material for: Neuronal ceroid lipofuscinosis in the South American-Caribbean region: An epidemiological overview
Source: Front Neurol. 2022 Aug 12;13:920421. doi: 10.3389/fneur.2022.920421 (PMC9412946; doi:10.3389/fneur.2022.920421)
Supplement: Supplementary file 7 [file Data_Sheet_1.PDF]

Title	Authors	Year
“Atypical” phenotypes of neuronal ceroid lipofuscinosis: the Argentine experience in the genomic era	Pesaola, *et al.*	2021
Abstracts of the 14th International Conference on Neuronal Ceroid Lipofuscinoses (batten disease) & 2nd International Patients Organization Meeting, October 22-26, 2014, Córdoba, Argentina	NCL 2014	2014
An integrated strategy for the diagnosis of neuronal ceroid lipofuscinosis types 1 (CLN1) and 2 (CLN2) in eleven Latin American patients	Kohan, *et al.*	2009
Cerliponase Alfa for the Treatment of Atypical Phenotypes of CLN2 Disease: A Retrospective Case Series	Wibbeler, *et al.*	2021
Ceroid-Lipofuscinosis in a Cocker Spaniel Dog	Minatel, *et al.*	2000
Childhood neuronal ceroid-lipofuscinoses in Argentina	Taratuto, *et al.*	1995
Clinical and electroencephalographic aspects of late infantile neuronal ceroid lipofuscinosis	Caraballo, *et al.*	2005
Clinical, biochemical and molecular characteristics of five patients with late infantile neuronal ceroide lipofucsinosis type (CLN2 disease) phenotype clasical and atypical	Atanacio, *et al.*	2019
CLN2 disease (neuronal ceroid lipofuscinosis type 2): Experience in the real world with cerliponase alfa intracerebroventricular enzyme replacement therapy in a public hospital in Cordoba, Argentina	Seratti, *et al.*	2018
CLN8 disease caused by large genomic deletions	Beesley, *et al.*	2016
CLN8 Mutations Presenting with a Phenotypic Continuum of Neuronal Ceroid Lipofuscinosis—Literature Review and Case Report	Badura-Stronka, *et al.*	2021
Congenital CLN8 disease of neuronal ceroid lipofuscinosis: a novel phenotype	Pesaola, *et al.*	2019
Diagnosis of neuronal ceroid lipofuscinosis type 2 (CLN2 disease): Expert recommendations for early detection and laboratory diagnosis	Fietz, *et al.*	2016
Ethical issues with artificial nutrition of children with degenerative brain diseases	Kohlschütter, *et al.*	2015
Future perspectives: Moving towards NCL treatments	Cotman, *et al.*	2015
Gene symbol: CLN5. Disease: neuronal ceroid lipofuscinosis, Finnish variant	Kohan, *et al.*	2008
Gene symbol: CLN6. Disease: Neuronal ceroid lipofuscinosis, late Infantile	Cismondi, *et al.*	2008
Guidelines for incorporating scientific knowledge and practice on rare diseases into higher education: neuronal ceroid lipofuscinoses as a model disorder	Cismondi, *et al.*	2015
Guidelines on the diagnosis, clinical assessments, treatment and management for CLN2 disease patients	Mole, *et al.*	2021
Intermediate phenotype of ATP13A2 mutation in two Chilean siblings: Towards a continuum between parkinsonism and hereditary spastic paraplegia	Miranda, *et al.*	2020
Is the ketogenic diet effective in specific epilepsy syndromes?	Nangia, *et al.*	2012
Late-onset childhood neuronal ceroid lipofuscinosis: Early clinical and electroencephalographic markers	Beltrán, *et al.*	2018
Management of CLN1 Disease: International Clinical Consensus	Augustine, *et al.*	2021
Management Strategies for CLN2 Disease	Williams, *et al.*	2017
Neuronal ceroid lipofuscinoses: description of 14 cases	Troncoso, *et al.*	2005
Neuronal Ceroid Lipofuscinosis (Batten Disease) in Latin America - an update	Noher de Halac & Dodelson de Kremer.	2005
Neuronal ceroid lipofuscinosis type CLN2: a new rationale for the construction of phenotypic subgroups based on a survey of 25 cases in South America	Kohan, *et al.*	2013
Palmitoyl Protein Thioesterase1 (PPT1) and Tripeptidyl Peptidase-I (TPP-I) are expressed in the human saliva. A reliable and non-invasive source for the	Kohan, *et al.*	2005

diagnosis of infantile (CLN1) and late infantile (CLN2) neuronal ceroid lipofuscinoses		
Position of Experts Regarding Follow-Up of Patients with Neuronal Ceroid Lipofuscinosis-2 Disease in Latin America	Guelbert, *et al*.	2020
Revealing the clinical phenotype of atypical neuronal ceroid lipofuscinosis type 2 disease: Insights from the largest cohort in the world	Lourenço, *et al*.	2021
Spectrum of CLN6 mutations in variant late infantile neuronal ceroid lipofuscinosis	Sharp, *et al*.	2003
The neuronal ceroid lipofuscinoses program: A translational research experience in Argentina	Kohan, *et al*.	2015
The neuronal ceroid lipofuscinosis-related protein CLN8 regulates endo-lysosomal dynamics and dendritic morphology	Pesaola, *et al*.	2021
The Parkinson-associated human P5B-ATPase ATP13A2 modifies lipid homeostasis	Marcos, et al.	2019
The Parkinson-associated human P5B-ATPase ATP13A2 protects against the iron-induced cytotoxicity	Rinaldi, *et al*.	2015
The strategic function of the P5-ATPase ATP13A2 in toxic waste disposal	Tezanos Pinto & Adamo	2018
Therapeutic approaches to the challenge of neuronal ceroid lipofuscinoses	Kohan, *et al*.	2011
Ultrastructural analyses in a case series of 34 progressive encephalophaties	Jardim, *et al*.	2005
A Case Report on the Challenging Diagnosis of Neuronal Ceroid Lipofuscinosis Type 2 (CLN2)	Nunes, *et al*.	2020
A new phenotype associated with homozygous GRN mutations: complicated spastic paraplegia	Faber, *et al*.	2017
Análise quantitativa da deglutição orofaríngea em indivíduo gastrostomizado com Lipofuscinose Ceróide Neuronal: relato de caso	Marcondes Natel Sales, *et al*.	2013
ATP13A2 missense mutations in juvenile parkinsonism and young onset Parkinson disease	Di Fonzo, *et al*.	2007
Atypical neuronal ceroid lipofuscinosis type 2 (CLN2 disease): A case report	Curiatis Mendes, *et al*.	2020
Diagnosis of metabolic diseases of the nervous system in children through ultrastructural analysis of non cerebral tissue	Rosemberg	1998
Dysregulation of autophagy as a common mechanism in lysosomal storage diseases	Seranova, *et al*.	2017
Electron microscopical study in neurodegenerative diseases in infancy	Bleggi Torres, *et al*.	1997
Juvenile neuronal ceroid-lipofuscinosis: clinical and molecular investigation in a large family in Brazil	Valadares, *et al*.	2011
Neuronal ceroid lipofuscinoses: a clinical and morphological study of 17 patients from southern Brazil	Puga, *et al*.	2000
Neuronal ceroid lipofuscinosis: clinical and neuroradiological findings	Gama, *et al*.	2007
Neuropathologic Findings in a Patient With Juvenile-Onset Levodopa-Responsive Parkinsonism Due to ATP13A2 Mutation	Chien, *et al*.	2021
Pitfalls in the clinical and electroencephalographic diagnosis of ceroid lipofuscinosis	Vasques, *et al*.	2005
Retinal function in patients with the neuronal ceroid lipofuscinosis phenotype	Quagliato, *et al*.	2017
Clinical spectrum of Kufor-Rakeb syndrome in the Chilen kindred with ATP13A2 mutations	Behrens, *et al*.	2010
Hereditary parkinsonism with dementia is caused by mutations in ATP13A2, encoding a lysosomal type 5 P-type ATPase	Ramirez, *et al*.	2006
Impaired temporo-occipital blood flow in an atypical CLN1 case with late infantile onset and granular osmiophilic deposits	Philippart, *et al*.	2001
A CLN5 mutation causing an atypical neuronal ceroid lipofuscinosis of juvenile onset	Trujillo, *et al*.	2005
Exome sequencing is an efficient tool for variant late-infantile neuronal ceroid lipofuscinosis molecular diagnosis	Patiño, *et al*.	2014

Lipofuscinosis ceroidea neuronal 6 (enfermedad Kufs tipo A): Reporte de caso en Colombia	Gonzalez Pabon, *et al*.	2021
Real world effectiveness of cerliponase alfa in classical and atypical patients. A case series	Espitia Segura, *et al*.	2021
Chromosomal localization of two genes underlying late-infantile neuronal ceroid lipofuscinosis	Haines, *et al*.	1998
Neuronal Ceroid Lipofuscinosis Type 6 (CLN6) clinical findings and molecular diagnosis: Costa Rica's experience	Badilla-Porras, *et al*.	2022
The gene mutated in variant late-infantile neuronal ceroid lipofuscinosis (CLN6) and in nclf mutant mice encodes a novel predicted transmembrane protein	Wheeler, *et al*.	2002
Neuronal ceroid lipofuscinosis. Type 6 late infantile variant in two compound heterozygous siblings with novel mutations	Bravo-Oro, *et al*.	2021
Neuronal ceroid lipofuscinoses. Clinical experience in 5 Mexican families	Ruiz Garcia, *et al*.	2005
Late-Infantile Neuronal Ceroid Lipofuscinoses (Jansky-Bielschowsky Disease): a Study of a Series of Cases	Ortiz, *et al*.	2014
Characterization of neuronal ceroid lipofuscinosis in Venezuelan children	Peña, *et al*.	2004
Expert recommendations for the laboratory diagnosis of neuronal ceroid lipofuscinosis type 2 (CLN2 disease): Diagnostic algorithm and best practice guidelines for a timely diagnosis	Giuliani, *et al*.	2016
Mutations in a novel CLN6-encoded transmembrane protein cause variant neuronal ceroid lipofuscinosis in man and mouse	Gao, *et al*.	2002
Neurological aspects of ceroid-lipofuscinoses	Peña, *et al*.	2000
Serial MRI findings in the Costa Rican variant of neuronal ceroid-lipofuscinosis	Peña, *et al*.	2001
Tripeptidyl peptidase 1 in patients with late infantile neuronal ceroid lipofuscinosis	Miranda Contreras, *et al*.	2012
